# Awareness of pregnancy induced hypertension among pregnant women in Tigray Regional State, Ethiopia

**DOI:** 10.11604/pamj.2020.35.71.19351

**Published:** 2020-03-11

**Authors:** Abadi Kidanemariam Berhe, Abiodun Olatunbosun Ilesanmi, Christopher Odianosen Aimakhu, Afework Mulugeta Bezabih

**Affiliations:** 1 Pan African University Institute for Life and Earth Sciences (PAULESI), University of Ibadan, Ibadan, Nigeria; 2 College of Medicine and Health Sciences, Adigrat University, Tigray, Ethiopia; 3 Department of Obstetrics and Gynaecology, College of Medicine, University College Hospital, University of Ibadan, Ibadan, Nigeria; 4 Department of Obstetrics and Gynaecology, College of Medicine, University College Hospital, University of Ibadan, Ibadan, Nigeria; 5 School of Public Health, College of Health Sciences, Mekelle University, Tigray, Ethiopia

**Keywords:** Awareness, pregnancy-induced hypertension, pregnant women, Tigray, Ethiopia

## Abstract

**Introduction:**

Pregnancy-induced hypertension is among the leading cause of maternal mortality in Tigray regional state, Ethiopia. However, there was no study in this study area about awareness of pregnancy induced hypertension among pregnant women. Therefore, the aim of this study was to assess awareness of pregnancy induced hypertension among pregnant women.

**Methods:**

A cross-sectional study design was conducted on a total of 798 pregnant women attending antenatal care in general hospitals of Tigray Regional State. Data were collected from February to November 30, 2018. Binary logistic regression analysis was used to determine factors associated with poor awareness and p-values < 0.05 was considered as statistically significant.

**Results:**

A total of 792 pregnant women were included in this study with a response rate of 99.2%. In this study, 41.8% of pregnant women were having poor awareness of pregnancy-induced hypertension. Primigravida, women with no formal education, women with the lowest wealth status and occupation of a housewife were significantly associated with poor awareness. Additionally, there was a significant difference in the mean score of awareness of pregnancy-induced hypertension between normotensive women and women with pregnancy-induced hypertension (Mean score difference (95% CI) = 1.90(1.35, 2.45), t = 6.75, df = 790, p < 0.001)).

**Conclusion:**

A high proportion of pregnant women had poor awareness on pregnancy-induced hypertension. Health care providers should improve awareness of pregnant women about pregnancy-induced hypertension in antenatal care clinics and at a community level with a special focus of awareness on primigravida women, women with no formal education, women with lowest wealth status and housewives.

## Introduction

Pregnancy-induced hypertension (PIH) is defined as new hypertension that appears at 20 weeks or more gestational age with or without proteinuria[1-3]. Hypertension during pregnancy is defined as a sustained systolic BP ≥ 140 mmHg or diastolic BP ≥ 90 mmHg [4]. Globally, pregnancy-induced hypertension is a significant public health threat both in developed and developing countries contributing to high maternal and perinatal morbidity and mortality [5]. According to World Health Organization (WHO) systematic analysis, hypertensive disorders of pregnancy attributed to 14% of maternal mortality and it is the second leading cause of maternal death after hemorrhage in sub-Saharan Africa which accounts for 16.0% of maternal mortality [6]. Similarly, WHO review identified hypertensive disorders of pregnancy were annually responsible for about 25,000 maternal deaths in Africa, 22,000 maternal deaths in Asia, 3,800 maternal deaths in Latin America and the Caribbean and 150 maternal deaths in industrialized countries[7, 8]. Studies conducted on the global impact of preeclampsia and eclampsia showed that preeclampsia was associated with higher rates of preterm delivery, small for gestational age babies, stillbirth and low birth weight[9]. Fetal morbidity and mortality increase substantially in women with preeclampsia and it is one of the leading causes of stillbirths and neonatal deaths [10, 11]. The prevalence of pregnancy-induced hypertension in Ethiopia ranges from 2.2% up to 18.3% [12-17]. Hypertensive disorders of pregnancy are among the five leading causes of maternal deaths in Ethiopia which account for 19% of deaths [18]. There are improvement in antenatal care (ANC) services in the last decade, according to the Ethiopian demographic and health survey (EDHS) report in Ethiopia but still, only 75% of pregnant women had their blood pressure measured, and 66% had urinetested[19, 20]. A study conducted in the current study area, Tigray regional state, Ethiopia revealed that pregnancy induced hypertension is among the three most common obstetric causes of maternal mortality [21]. Recent evidence suggests that the presence of complications related to hypertension disorder of pregnancy was the result of inadequate knowledge; negative attitude towards hypertension in pregnancy and lack of preventive practice [22]. In addition, studies showed that women with a good awareness of pregnancy induced hypertension were more likely to promptly report symptoms and seek health care [23, 24]. Poor awareness of pregnant women is one of the potential factors for delay in seeking care and decision and a bottleneck for early diagnosis and management of critical illnesses. However, there was no evidence about the awareness of pregnant women regarding pregnancy induced hypertension in Tigray Regional State, Ethiopia. Therefore, the findings of this study would primarily contribute to the existing limited evidence in this area and also have a great significance for program coordinators and health care facilities to design and implement effective strategies for the prevention and early management of pregnancy-induced hypertension. In addition, the data will be used as a baseline for other researchers who want to investigate further studies in this area. Hence the aim of this study was to assess awareness of pregnancy induced hypertension among pregnant women in general hospitals of Tigray Region State, Ethiopia.

## Methods

**Study setting and design:** this study was conducted in Tigray regional state. Tigray Regional State is the northernmost of the nine Regional States of Ethiopia. It is bordered by Eritrea to north, Sudan to west, Afar region to the east and Amhara region to the south.The total projected population of the region for 2017 was 5,396,235; of which 2,654,947 are males and 2,741, 287 females [25]. The reproductive age group (15-49 years) comprises of 23.5% of the population. There were 173,892 total expected pregnancies which give a pregnancy rate of 3.5%. According to the Regional Health Bureau Report, there are fifteen general hospitals in the region. The study was conducted in eight general hospitals, which are geographically distributed over the entire Tigray Regional State, namely; Lemlem Carl, Mekelle, Adigrat, Wukro, Adwa, Saint Marry Axum, Suhul Shre, and Kahsay Abera Humera hospitals. A cross-sectional study design was used to assess awareness of pregnancy induced hypertension. The study period was from February to November 30, 2018.

**Study population:** the study population was all pregnant mothers attending antenatal care in the selected eight hospitals during the study period. Critically ill women who could not respond to the interview were excluded from this study.

**Sample size and sampling technique:** a total of 798 study participants were included in this study. The calculated sample size was proportionally allocated to eight selected general hospitals based on the number of pregnant mothers attending antenatal care in each hospital. Participants were selected using a systematic sampling method.

**Data collection instruments and methods:** a structured questionnaire containing information on socio-demographic data and awareness on pregnancy induced hypertension were used for assessment. The questionnaire was prepared by reviewing different literatures like published works, research articles, EDHS related to the topic and then adapted to the local context to maintain validity [14, 20, 26-34]. Overall the questionnaire was initially prepared in the English version and translated to Tigrigna (local language) version by language expert and back converted again to English version by another person to check the consistency. Midwives and nurses having experience in research assistance were involved in the data collection and supervision of the study respectively. Three days of training was provided for all data collectors and supervisors before the actual data collection period on the objectives of the study, contents of the data collection instrument, ethical issues and interviewing techniques. Pretest of the data collection instrument was conducted on 5% of the total sample size in two selected hospitals (Abiadi and Alamata hospitals) of Tigray Regional State which were not included in the study. An unclear idea, the time needed for the interview, other technical related problems on the data collection instrument was corrected based on the result of the pretest. Daily close supervision and spot checks of the filled-in questionnaire were done by the field supervisor deployed with the data collectors. The overall data collection process was coordinated and supervised by supervisors and the principal investigator.

**Operational definition:** pregnancy-induced hypertension (PIH): is an increment of SBP of at least 140mmHg and/or DBP of at least 90mmHg with or without proteinuria after 20 weeks of gestation in previously normotensive women. There were 14 questions for the assessment of awareness on pregnancy induced hypertension which addressed the definition of PIH, prevention of PIH, PIH signs/symptoms, PIH complications & risks and management of PIH. The correct answer was given a score point of one and the wrong answer was given a score of zero. A total awareness score was computed by summing correct answers. **Good awareness:** if the pregnant women answered the awareness questions above or equal to the mean score ( ≥7 scores). **Poor awareness:** if the pregnant women answered the awareness questions below the mean score(< 7 scores).

**Data analysis and management:** statistical analysis was done using SPSS version 21.0. Descriptive statistics frequencies and mean, standard deviation and the percentage were used to describe the study population. Independent t-tests were used to evaluate the difference in mean awareness scores of pregnancy induced hypertension between women with PIH and normotensive women. Multivariable logistic regression analysis was used to determine factors associated with poor awareness by avoiding confounders. Variables significant at p-value < 0.05 in the univariate model were moving to a multivariable analysis model to identify independent factors. In the final model p-value < 0.05 was considered as statistically significant. Model goodness of fit for logistic regression was assessed using the Hosmer-Lemeshow goodness of fit test.

**Ethical consideration:** ethical approval and clearance for the study obtained from University of Ibadan, University College Hospital institutional ethical review committee (Ref. number NHREC/05/01/2008a). Official letter of support to study areas was also received from Tigray Regional Health Bureau to selected study areas. Confidentiality and anonymity of study participants were ensured. Written informed consent was sought from the participants prior to data collection.

## Results

**Socio-demographic characteristics of study participants:** a total of 792 pregnant women were included in this study with a response rate of 99.2%. The mean age of study participants was 27.3 years with a standard deviation of 5.7 years. Majority of the participants were residing in urban 621 (78.4%) and 94 (11.9%) of the participants had no formal education. Larger proportions 240(91.3%) of mothers were married. With regard to the occupation majority of study participants (46.5%) were housewives. Concerning wealth status 156 (19.7%) of the participants were in the lowest wealth quantile. In addition majority of the participants were Orthodox Christians 680 (85.9%) in religion. Detail socio-demographic characteristics of participants indicated in Table 1.

**Table 1:** Socio-demographic characteristics of pregnant women attending antenatal care in general hospitals of Tigray Regional State, Ethiopia, 2018 (n=792)

Variable	Frequency	Percent
**Maternal age**		
≤ 19 years	46	5.8
20-34 years	639	80.7
≥ 35 years	107	13.5
**Residence**		
Urban	621	78.4
Rural	171	21.6
**Maternal educational status**		
No formal education	94	11.9
Primary	220	27.8
Secondary	252	31.8
Diploma and above	226	28.5
**Religion**		
Orthodox	680	85.9
Catholic	33	4.2
Muslim	66	8.3
Protestant	13	1.6
**Maternal occupation**		
House wife	368	46.5
Farmer	70	8.8
Private business	155	19.6
Governmental/ NGO employee	199	25.1
**Marital status**		
Married	723	91.3
Single	69	8.7
**Wealth index**		
Lowest	156	19.7
Second	158	19.9
Middle	156	19.7
Fourth	162	20.5
Highest	152	19.2

**Figure 1:**
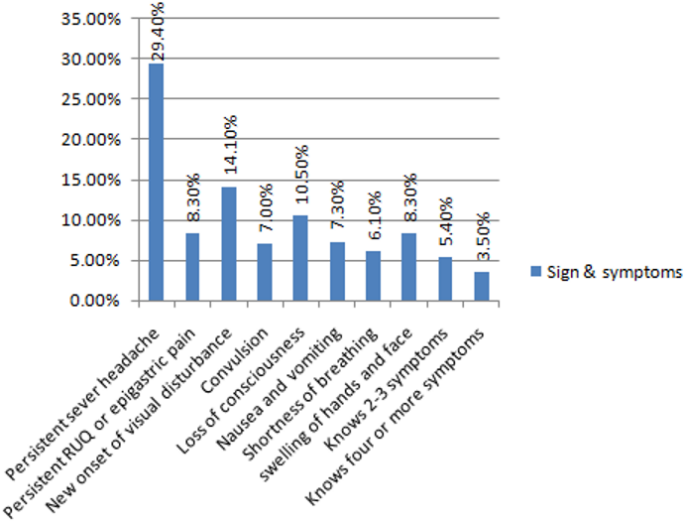
Signs and symptoms of pregnancy induced hypertension identified by pregnant women attending antenatal care in Tigray Regional State, Ethiopia, 2018

**Awareness of pregnancy induced hypertension among pregnant women:** from the participants involved in this study 459 (58.0%) ever heard about pregnancy induced hypertension. Most of the participants heard about pregnancy induced hypertension from health care providers 203 (44.3 %) and friends 104 (22.7%) as a source of information. More than three fourth of the participants (78.0%) did not know when pregnancy induced hypertension starts to occur. Only 323 (40.8%) of women were thinking of PIH conditions heal after delivery or a few weeks after delivery. Similarly, 304 (38.4%) of women were thinking as any pregnant woman is at risk for PIH. In addition; 308 (38.9%) of the participants identified at least one sign and symptom of pregnancy induced hypertension (Table 2). The most common signs and symptoms of pregnancy induced hypertension identified by the participants were persistent headache 92 (29.4%), new onset of visual disturbance 44 (14.1%), loss of consciousness 33 (10.5%) and persistent right upper quadrant pain or epigastric pain 26 (8.3%). In addition, 17 (5.4%) of participants identify two or more signs and symptoms of pregnancy-induced hypertension (Figure 1). The finding of this study showed that 331 (41.8%) of pregnant women were having poor awareness of pregnancy induced hypertension. Only 470 (59.3%) of participants were thought that pregnancy induced hypertension can be preventable. On the other hand, 355 (44.8%) of pregnant women did not think regular exercise can prevent pregnancy induced hypertension. In addition, 603 (76.1%) of pregnant women were aware as regular antenatal care check-ups by health care providers can be used for early detection of PIH. Almost two-thirds of participants 560 (70.7%) were also aware that pregnancy induced hypertension can be treated. Similarly, 441 (55.7%) of pregnant women were aware that excess body weight loss before conception can prevent pregnancy-induced hypertension. Approximately one- fifth (22.5%) of pregnant women knew at least one management of pregnancy induced hypertension. Among those participants having the knowledge on the management of pregnancy-induced hypertension, majority (46.6 %) of them knew anti-hypertensive drugs as management of PIH but anticonvulsant was the least identified management of PIH (1.7 %) among participants (Table 3).

**Table 2:** Awareness of pregnancy induced hypertension among pregnant women attending antenatal care in general hospitals of Tigray Regional State, Ethiopia, 2018 (n=792)

Variables	Frequency	Percent (%)
**Women ever heard of pregnancy induced hypertension**		
Yes	459	58.0%
No	333	42.0%
**Source of information about pregnancy induced hypertension**		
Health facility	203	44.3%
Media	54	11.8%
Friends	104	22.7%
Women development group	39	38.5%
Personal experiences	9	2.0%
Other	3	0.7%
More than two sources	46	10.0%
**When can pregnancy induced hypertension starts to occur**		
Before pregnancy	44	5.6%
During pregnancy after 20 weeks of gestation	175	22.1%
Before and during pregnancy	98	12.4%
I did not know	475	60.0%
**Women thinking of PIH conditions heal after delivery or a few weeks after delivery**		
Yes	323	40.8%
No	469	59.2%
**Thinking of any pregnant woman, even a healthy one, is at risk for PIH**		
Yes	304	38.4%
No	488	61.6%
**How serious of a health issue do you think PIH is**		
Very serious	324	40.9%
Serious	438	55.3%
Not at all serious	30	3.8%
**Do you know any sign and symptoms of PIH**		
Yes	308	38.9%
No	484	61.1%

**Table 3:** Awareness on prevention and management of pregnancy induced hypertension among pregnant women attending antenatal care in general hospitals of Tigray regional state, Ethiopia, 2018 (n=792)

Variable	Frequency	Percent (%)
**Pregnancy-induced hypertension can be prevented**		
Yes	470	59.3%
No	322	40.7%
**Regular exercise can prevent pregnancy-induced hypertension**		
Yes	465	58.7%
No	327	41.3%
**Regular antenatal care check-up by health care provider can be used for early detection of PIH**		
Yes	603	76.1%
No	189	23.9%
**Losing excess body weight before conception can prevent PIH**		
Yes	441	55.7%
No	351	44.3%
**Appropriate actions against PIH associated symptoms**		
Go to health facility	710	89.9%
Lie down	14	1.8%
Wait one day to see progress	36	4.6%
Go to traditional healers	26	3.3%
Other	4	0.5%
**Pregnancy induced hypertension can be treated**		
Yes	560	70.7%
No	232	29.3%
**Frequent prenatal check-up for blood pressure is important**		
Yes	588	74.2%
No	204	26.8%
**know any management of pregnancy-induced hypertension**		
Yes	178	22.5%
No	614	77.5%
**Type of PIH management identified by the women**		
Conservatively	54	30.3%
Antihypertensive	83	46.6%
Anticonvulsant	3	1.7%
Anti-HTN and anticonvulsant	25	14.1%
All	13	7.3%

**Figure 2:**
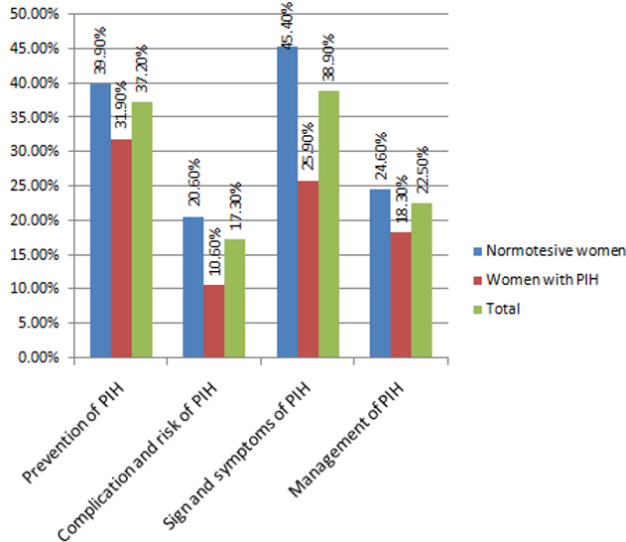
Participants correctly answering the questionnaire by subareas of awareness on pregnancy induced hypertension among pregnant women with PIH and normotensive women in Tigray Regional State, Ethiopia, 2018

**Comparison of awareness on pregnancy induced hypertension among women with PIH and normotensive women attending antenatal care:** findings of this study showed that from the overall fourteen awareness questions, the mean score (std) of correct responses of awareness among normotensive women and women with PIH was 8.2(3.7) and 6.3(3.8) respectively. This finding indicates a significant difference in the mean score of awareness questions on pregnancy induced hypertension between normotensive women and women with PIH (p < 0.001). The result of this study showed that normotensive participants had a better level of awareness compared to women with pregnancy-induced hypertension. In this study, 39.9% of normotensive pregnant women and 31.9% % of women with PIH have identified prevention techniques of PIH (Figure 2).

**Factors associated with awareness of pregnancy induced hypertension among pregnant women attending antenatal care:** in bivariate logistic regression analysis maternal age, place of residence, maternal educational status, maternal occupation, wealth status and number of gravida were found as a significant factors but after controlling for the effects of potentially confounding variables using multivariable logistic regression analysis only maternal educational status, maternal occupation, wealth status and number of gravida were found as a significant factors associated with poor awareness of pregnancy-induced hypertension among pregnant women attending antenatal care in general hospitals of Tigray Regional State, Ethiopia (Table 4).

**Table 4:** Factors associated with awareness of pregnancy induced hypertension among pregnant women attending antenatal care in general hospitals of Tigray Regional State, Ethiopia, 2018 (n=792)

Variable	Awareness of PIH		COR (95% CI)	AOR (95% CI)
**Maternal age**	**Good (%)**	**Poor (%)**		
≤ 19 years	20 (4.3%)	26 (7.9%)	0.53(0.28,0.96) *	1.1 (0.5, 2.1)
20-34 years	379(82.2%)	260 (78.5%)	1	1
≥ 35 years	62 (13.4%)	45 (13.6%)	0.94 (0.62,1.43)	1.3 (0.7, 2.2)
**Residence**				
Urban	394 (85.5%)	227 (68.6%)	1	1
Rural	67 (14.5%)	104 (31.4%)	0.37 (0.26,0.53) ***	1.1 (0.6, 1.6)
**Maternal educational status**				
No formal education	23 (5.0%)	71 (21.5%)	1	1
Primary	110 (23.9%)	110 (33.2%)	3.10 (1.80,5.29) ***	2.9 (1.6, 5.3) ***
Secondary	151 (32.8%)	101 (30.5%)	4.62 (2.71,7.81) ***	3.1 (1.6, 5.8) ***
College and above	177 (38.4%)	49 (14.8%)	11.15(6.33,19.65) ***	4.9 (2.3, 10.3) ***
**Maternal occupation**				
House wife	178 (38.6%)	190 (57.4%)	1	1
Farmer	24 (5.2%)	46 (13.9%)	0.55(0.32, 0.95) *	1.3 (0.6, 2.5)
Private business	103 (22.3%)	52(15.7%)	2.11 (1.43, 3.12) ***	1.2(0.8, 1.9)
Governmental/ NGO employee	156 (33.8%)	43 (13.0%)	3.87 (2.61, 5.74) ***	1.7 (1.1, 2.9) *
**Wealth index**				
Lowest	88(19.3%)	173(52.6%)	0.16 (0.11,0.24) ***	0.3 (0.2, 0.4) ***
Middle	169(37.1%)	93(28.3%)	0.58 (0.39,0.85) **	0.7 (0.5, 1.1)
Highest	198 (43.5%)	63 (19.1%)	1	1
**Number of gravida**				
1	141 (30.6%)	135 (40.8%)	0.64 (0.47,0.86) **	0.6 (0.4, 0.8) **
≥ 2	320 (69.4%)	196 (59.2%)	1	1

Reference group=1,***P<0.001,**P<0.01, *p<0.05

## Discussion

The purpose of this study was to assess the level of awareness regarding pregnancy induced hypertension and associated factors among pregnant women attending antenatal care in general hospitals of Tigray Regional State, Ethiopia. From the participants involved in this study, 58.0% (95% CI: 54.4%, 61.4%) ever heard about pregnancy induced hypertension. The finding of this study was lower than a study conducted in Tamale Metropolis and Sunderlal Hospital, Varanasi, India [33, 35] but higher than a study conducted in Kelantan, Malaysia [36]. This difference might be due to the differences in access to health services, mass media, and methods of information distribution among the countries. The major source of information to hear about pregnancy induced hypertension in this study was health facilities 203 (44.3 %). This finding was similar with a study done in Southwest Nigeria which indicated hospitals were a major source of information on pregnancy-induced hypertension but incongruent with the study done in a selected hospital, Dehradun, Uttarakhand, India which showed 50% of them got information from televisions [37]. This might be related to the differences in socioeconomic status, access to television and electricity. The findings of this study revealed that 41.8% (95% CI: 38.3%, 45.3%) of pregnant women were having poor awareness of pregnancy induced hypertension. This finding was higher than a study conducted in Bangalore [38], Southwest Nigeria [37], Adeoyo Maternity Hospital, Yemetu Ibadan, Nigeria [39], Karaikal India [40]. The possible reason could be the difference in the literacy level of pregnant mothers and difference access to information, education, and communication. More than three fourth of the participants (78.0%) did not know when pregnancy induced hypertension starts to occur. This result was lower than the study conducted in Bangalore [38]. This might be also due to the difference in access to health services and the participant's level of literacy. Only 38.4% of pregnant women believed that any pregnant woman is at risk of PIH. This finding was lower than a study conducted in south Nigeria which showed that 58.0% of participants believed that any pregnant women can develop pregnancy induced hypertension [37]. Such believe of pregnant women affects their regular antenatal care check-ups for blood pressure if they consider themselves as they are at risk of developing pregnancy induced hypertension.

Overall 38.9% (95%CI: 35.5%, 42.4%) of the participants knew at least one sign and symptom of pregnancy induced hypertension. This result was lower than a study conducted in Tamale Metropolis (49.8%) [33]. The possible reason for this variation could be the difference in literacy level and socio-economic status. The most common sign and symptoms of pregnancy induced hypertension identified by the participants in this study were persistent headache 29.4%, new onset of visual disturbance 14.1% and loss of consciousness 10.5%. Hence, the lower awareness of pregnancy induced hypertension will affect the early health-seeking behavior of pregnant women. Therefore, it is important to strengthen health education regarding pregnancy induced hypertension in all health facilities during antenatal care. Four hundred seventy-three (59.7%) participants were aware that pregnancy induced hypertension can be prevented. Despite of this, 44.8% of pregnant women did not believe regular exercise can prevent pregnancy induced hypertension. This finding was lower than the study conducted in Khartoum State, Sudan [41]. In addition, 76.1% of pregnant women were aware that regular antenatal care check-ups by health care providers can be used for early detection and prevention of PIH. This finding was lower than a study done in Bangalore which indicated 86.7% of participants believed that regular antenatal care check-ups of blood pressure can prevent PIH and its complications[38] and 84% in Nigeria [42]. Almost two-thirds (70.7%) of participants were aware that pregnancy induced hypertension can be treated. Similarly, 55.7% of pregnant women were aware that losing excess body weight before conception can prevent pregnancy induced hypertension. This result was consistent with the study conducted in Southwest Nigeria [37]. Approximately one- fifth (22.5%) of pregnant women knew at least one management method of pregnancy induced hypertension. This result was lower than a study conducted in Tanzania and Sudan [41,43]. Among those participants having knowledge on the management of pregnancy induced hypertension, majority (46.6 %) of them knew anti-hypertensive drugs as management of PIH. In this study, the majority (89.9%) of the participants believed going to a health facility for care was an appropriate action to take during the development of signs and symptoms associated with PIH. This finding was in line with a study done at Sunderlal Hospital, Varanasi, India[35].

Findings of this study showed that a significant difference in the mean score of awareness on pregnancy-induced hypertension between normotensive pregnant women and women with PIH (Mean score difference (95% CI) = 1.90 (1.35, 2.45), t = 6.75, df = 790, P < 0.001)) were occurred. The result of this study showed normotensive participants had a better level of awareness compared to women with PIH. This could be due to the poor counseling of health care providers in the antenatal care clinic about PIH, and/or variation in educational status and residence between the two groups in this study (normotensive pregnant women had better education status compared to women with PIH, and more normotensive pregnant women were from urban residence compared to women with PIH). Regarding the factors associated with awareness of PIH, findings of this study showed that mothers with college and above educational levels were 4.9 times more likely to be aware of pregnancy induced hypertension compared to women with no formal education. This result was consistent with a study done in Zabol, Iran, and Belgaum, Karnataka, India as well as with a study conducted in Utah [44-46]. It is known that educated mothers have better access to information, education and communication (IEC) from different media like internet, books, Television, etc. In addition, pregnant women with the lowest wealth status showed a poor level of awareness regarding PIH compared to pregnant women with the highest wealth status. This finding was in line with a study conducted in Karnataka, India which showed mothers with low income were associated with poor knowledge scores [45]. The reasons might be mothers with low wealth status have less access to mass media, transportation, and poor health-seeking behavior compared to women with the highest wealth status, this can lower the level of awareness on pregnancy-induced hypertension.

Similarly, the result of this study revealed that pregnant mothers with governmental/NGO employees had 1.7 times more likely to be aware of PIH compared to those pregnant women with the occupation of housewives. This finding was similar to a study conducted in selected hospitals of Mangalore, India [47]. This might be related with the access of information and education; governmental/private employed pregnant women had better access in getting information on pregnancy care from their peers in their organization and have a better educational level compared to housewife pregnant mothers. Additionally, in this study primigravida women had 0.6 times less likely to be aware of PIH compared to pregnant women with two or more experience of pregnancy. This finding was congruent with a study done in selected hospitals of Mangalore, India [47]. This might be related to less experience primigravida women about pregnancy health and pregnancy-related risk compared to multigravida women. To our knowledge, this is the first study conducted on awareness of pregnancy induced hypertension in Tigray regional state, Ethiopia. As a limitation, the finding of this study did not represent to the general population since this study was conducted among pregnant women who came for antenatal care in hospitals. Further qualitative research will be beneficial to identify an in-depth understanding of mothers' views on pregnancy induced hypertension. However, this study will be used as a preliminary survey since there is no published data regarding the level of awareness among pregnant women specifically in Tigray Region State and generally in Ethiopia.

## Conclusion

In this study, a high proportion of pregnant women had poor awareness on pregnancy induced hypertension in general hospitals of Tigray Regional State. There was a significant difference in awareness of pregnancy induced hypertension among normotensive pregnant women and women with pregnancy-induced hypertension. Maternal educational status, maternal occupation, wealth status, and gravidity were the factors associated with awareness of pregnancy-induced hypertension among pregnant women in this study. Health care providers should strengthen the awareness of pregnant women about pregnancy-induced hypertension in antenatal care clinics and at the community level with a special focus of awareness to primigravida women, women with no formal education, women with lowest wealth status and housewife.

### What is known about this topic

- No previous published study on awareness of pregnancy induced hypertension among pregnant women in Tigray Regional State, Ethiopia;
- There is a high prevalence of pregnancy induced hypertension in Tigray Regional State and generally in Ethiopia;
- Pregnancy induced hypertension is among the top three leading causes of maternal mortality in Tigray Regional State, Ethiopia.

### What this study adds

- No previous published study on awareness of pregnancy induced hypertension among pregnant women in Tigray Regional State, Ethiopia;
- There is a high prevalence of pregnancy induced hypertension in Tigray Regional State and generally in Ethiopia;
- Pregnancy induced hypertension is among the top three leading causes of maternal mortality in Tigray Regional State, Ethiopia.

## Competing interests

The authors declare no competing interests.
